# Dosage Forms Suitability in Pediatrics: Acceptability of Analgesics and Antipyretics in a German Hospital

**DOI:** 10.3390/pharmaceutics14020337

**Published:** 2022-01-31

**Authors:** Viviane Klingmann, Thibault Vallet, Juliane Münch, Robin Stegemann, Lena Wolters, Hans-Martin Bosse, Fabrice Ruiz

**Affiliations:** 1 Department of General Pediatrics, Neonatology and Pediatric Cardiology, Medical Faculty, University Children’s Hospital Düsseldorf, Moorenstrasse 5, 40225 Düsseldorf, Germany; juliane.muench@med.uni-duesseldorf.de (J.M.); robin.stegemann@hhu.de (R.S.); lena.wolters@hhu.de (L.W.); hansmartin.bosse@med.uni-duesseldorf.de (H.-M.B.); 2 ClinSearch, 110 Avenue Pierre Brossolette, 92240 Malakoff, France; thibault.vallet@clinsearch.net

**Keywords:** pediatric drug formulation, drug administration, infants, toddlers, ibuprofen, paracetamol, analgesics, acceptability, swallowability, palatability

## Abstract

Although medicine acceptability is likely to have a significant impact on the patient’s adherence in pediatrics and therefore on therapy success, there is still little data even for common therapeutic areas. For analgesics/antipyretics, healthcare professionals face a wide variety of products and need knowledge to select the best adapted product for each patient. We investigated acceptability of those products most used at the University Children’s Hospital Düsseldorf, Germany. Based on 180 real-life observer reports of medicine intake, we used the acceptability reference framework to score acceptability of six distinct medicines. Both ibuprofen and paracetamol tablets, mainly used in adolescents, were positively accepted. This was not the case for the solution for injection of metamizole sodium. Regarding syrups, mainly used in children under 6 years of age, ibuprofen flavored with strawberry and provided with an oral syringe was positively accepted, while paracetamol flavored with orange and provided with a measuring cup was not. Suppository appeared to be an alternative to oral liquids in infants and toddlers with palatability and administration issues. Differences appeared to be driven by dosage forms and formulations. These findings improve knowledge on acceptability drivers and might help formulating and prescribing better medicines for children.

## 1. Introduction

In addition to the effectiveness of a drug, the product acceptability plays a decisive role: no matter how potent a drug may be, if the patient refuses to take it, it cannot develop its efficacy. Acceptability, which is the “overall ability and willingness of the patient to use and its care giver to administer the medicine as intended” [1], is thus an essential criterion for designing and prescribing medicines. This aspect is crucial in patients from the pediatric populations who require special considerations, as they cannot be regarded as small adults nor as a homogeneous group due to physical, metabolic, and psychological development. The European Medicine Agency’s (EMA) Pediatric Regulation 2007 aimed to increase the availability of pediatric dosage forms and pediatric trials, to improve the safety and compliance of medication administration in childhood [2]. Furthermore, the 2014 EMA guideline on pharmaceutical development of medicines for pediatric use emphasized that “evaluation of patient acceptability of a pediatric preparation should be an integral part of pharmaceutical and clinical development” [1].

To date, solid oral dosage forms (SODF) that were developed for adults are often used in pediatrics. Children’s inability to swallow SODF or inappropriate drug strength result in the crushing of tablets, or the opening of capsules, which may lead to dosing inaccuracies and affect bioavailability [3,4]. In addition, many drugs taste bitter and thus taste-masking is crucial to avoid aversiveness of medicine in children [5,6]. Acceptability appears to be a complex multi-faceted concept determined by the characteristics of both the patient (e.g., age, preexisting conditions, sociocultural background) and the medicine (e.g., swallowability, palatability, usability), as well as the context of use and the care giver [1]. Acceptability thus consists not only of the patient’s actual acceptance when administering the drug, but also of the external circumstances that lead to taking the drug. Despite significant improvement driven by new regulations, the knowledge on medicine acceptability in pediatrics is still fragmented. In recent years, several studies have been conducted on new child-directed oral dosage forms, among others by our study group [7,8,9,10,11,12]. However, in addition to developing new dosage forms, it is also important to know the acceptability of drugs currently on the market.

Medicines from the class of analgesics and antipyretics are very commonly used to treat pain and fever. Consequently, there are many dosage forms available on the market for ordinary drugs such as paracetamol (acetaminophen) or ibuprofen. For example, 235 medicinal products for paracetamol and 99 medicinal products for ibuprofen are/were available on the French market for the last three years [13]. The situation is similar in Germany, where 257 medicinal products for paracetamol and 262 medicinal products for ibuprofen are currently available on market [14]. There is a wide variety among these products in terms of route of administration (e.g., oral, parenteral, rectal), dosage form (e.g., tablet, capsule, orodispersible, effervescent tablet, sachet, oral solution), strength (from 100 mg to 1000 mg of paracetamol), or excipients (e.g., many different flavors and sweeteners for oral medicines). To deal with this diversity, healthcare professionals need relevant knowledge to select the best-adapted medicinal product, ensuring adequate acceptability for the concerned patient.

In 2019, a French study explored the acceptability of paracetamol dosage forms in vulnerable patients, including those of the older (≥65 years of age) and the pediatric (<18 years of age) populations [15]. Due to the limited number of medicinal products assessed in this primary study, further investigations were needed. Therefore, we used the same validated multivariate approach—the ClinSearch Acceptability Score Test^®^ (CAST) [15,16,17,18,19,20,21,22,23]—to explore acceptability of the analgesics/antipyretics most used in pediatrics at the University Children’s Hospital Düsseldorf.

## 2. Materials and Methods

### 2.1. Study Design, Setting, and Objective

This monocentric, cross-sectional, and observational study was conducted in University Children’s Hospital Düsseldorf (Düsseldorf, Germany) between August 2020 and June 2021. The Ethics Committee of the Medical Faculty of the Heinrich-Heine-University Düsseldorf gave a favorable opinion for the study on 23 July 2020 (no. 2020-962). The objective was to explore the acceptability of different medicinal products from the drug class of analgesics/antipyretics in different dosage forms and formulations in children aged newborn to 18 years.

### 2.2. Participants and Sample Size

According to German law, a signed consent of both parents was mandatory before starting any study related procedures. Where possible due to age, an assent of the patient was obtained. All eligible subjects under 18 years of age treated with one of the six following medicinal products were approached by a member of the research team and asked if they would like to participate on a voluntary basis, without any randomization:

- Paracetamol 500 mg Tablet (Hexal^®^)
- Ibuprofen 400 mg Tablet (Zentiva^®^)
- Paracetamol 40 mg/mL Syrup (Ratiopharm^®^)
- Ibuprofen 4% Syrup (Zentiva^®^)
- Paracetamol 125 mg Suppository (Stadapharm^®^)
- Novaminsulfon 1 g Solution for injection (Zentiva^®^)

These medicines were those from the drug class of analgesics/antipyretics most used at the University Children’s Hospital Düsseldorf.

Patients receiving intravenous medication where the intravenous device is already in situ—as insertion of such a device was considered as part of the acceptability evaluation—were not included in the study.

Thirty evaluations of the intake by individual patients of each medicine were necessary to get an acceptability score with a satisfactory precision using the CAST methodology [16,17]. The intake of only one of the six medicinal products was assessed per patient and consequently, 180 evaluable patients had to be included in the study.

### 2.3. Data Collection

Once enrolled in the study a standardized questionnaire was completed by a trained researcher observing the first administration of the medicine under investigation. The researcher reported the following observations: (1) the results of intake (the required dose was fully, partly, or not taken); (2) the patient´s reaction to the intake (positive, neutral, or negative on a 3-point facial hedonic scale); (3) the time hospital staff needed to prepare (from opening the packaging to having a required dose of medication ready to use, including all handling and modifications) and administer the required dose of medication (from a required dose of medication ready to use to the end of the intake), pooled and recoded as short (≤1’), medium (from 1′ to 2′30″), or long (>2′30″). In addition, the methods used to ease/achieve administration were reported resulting in 6 binary variables; (4) dividing the intake of a required dose which cannot be taken as a whole (e.g., successive sips of an oral liquid preparations, several tablets or pieces of tablet swallowed successively); (5) altering the use, such as modifying the dosage form (e.g., prescribed dose of tablet split into fractions or crushed into powder) or using another route/mode of administration (e.g., oral administration of an injectable solution); (6) using food/drink (e.g., mixing with the drug or taking before/after to mask the taste or ease swallowing); (7) using a device not provided with the medication (e.g., disposable spoon or oral syringe provided with another medicinal product); (8) promising a reward; (9) using restraint (i.e., the child was forced to take it). Each evaluation corresponded to a specific combination of one observed measure (e.g., the required dose was fully taken) for each of the nine aforementioned observational variables (e.g., the result of the intake).

Beyond the observer-reported outcomes describing acceptability, the researcher should also record information on the patient (e.g., age, sex, geographical regions of origin of both parents) and the prescribed treatments (e.g., the required dose and dosing frequency) from the patient’s medical record, as well as information on medicine use circumstances (e.g., time of administration).

### 2.4. Data Analysis

Acceptability scoring was performed using the acceptability reference framework: an intelligible model based on real-life observer-reported outcomes collected in an international acceptability study carried out since 2015 [15,16,17,18,19,20,21,22,23]. Multivariate analysis mined a large set of 3130 evaluations, comprised of those collected in this sub-study in Germany, and those previously collected using the same standardized questionnaire in eight other countries with various cultures (France, Norway, the United Kingdom, Poland, Morocco, India, Japan, Peru) in both domestic and hospital settings.

First a mapping process, multiple correspondence analysis, summarized the variability between all the evaluations—combinations of nine observed measures—and the key relationships between the observed measures themselves, into a low-dimensional Euclidian space: the three-dimensional (3D) acceptability map. Proximities on the 3D-map reflected similarities between elements: observed measures closed on the map were often selected together in the evaluations, while evaluations completed in a comparable manner converged on the map. The evaluations were positioned all over the 3D-map, between the ideal combination reflecting a medicine use without any problem and the combinations with the worst observed measures. There were 374 distinct combinations of observed measures illustrating the variability of usage observed in real-life conditions. Subsequently, evaluations were gathered into clusters according to Euclidean distances on the 3D-map, using hierarchical clustering on principal components and k-means consolidation. The clusters defined two coherent and meaningful acceptability profiles, which were described by the observed measures over-represented into their subset of evaluations. Therefore, the acceptability reference framework consisted of the 3D-map juxtaposing the ‘positively accepted’ and the ‘negatively accepted’ profiles, materialized by green and red areas on the map, respectively.

All the evaluations collected in this study were plotted on the 3D-map. The centroid (barycenter) of the evaluations of each studied medicinal products defined the medicine’s position on the acceptability map. Confidence ellipses surrounding each centroid for all dimension pairs (1–2, 1–3, and 2–3) defined an area containing its true position with 90% probability if the experiment were to be repeated. If a barycenter, along with the entire confidence ellipsis surrounding it, belonged to the ‘positively accepted’ profile the medicine was classified as positively accepted. Acceptability scores were significantly different if confidence ellipses did not overlap on the 3D-map. A minimum of 30 evaluations were required to obtain a reliable score with a satisfactory precision using the CAST methodology. Below this threshold we can only describe acceptability tendency.

The significance of the differences observed in terms of patients’ characteristics (e.g., age, sex) and observer-reported outcomes composing the acceptability scores were assessed using Pearson’s chi-squared test (χ^2^) or alternatively, Fisher’s exact test (F) when there were few observations for individual cells of the contingency table (less than expectation of 5 for 20%) or null expectation.

Data analysis was performed using R version 1.0.136^©^ (RStudio Team (2016). RStudio: Integrated Development for R. RStudio, Inc., Boston, MA, USA). The R packages FactoMineR [24] and missMDA [25] were used to perform multivariate analysis and to handle missing data, respectively.

## 3. Results

### 3.1. Study Subject

In this study, 180 evaluations were collected: 30 for each medicine of interest. The mean age of the patients was 8.5 years (SD = 6.2, range 0–17) and 45% were girls. Table 1 presents the characteristics of the patients stratified by medicinal product.

There was no significant difference in terms of sex (χ^2^: *p* = 0.27) and geographical regions of origin of parents (F: *p* = 0.3) between the subgroups of patients treated with the six medicines of interest. Both paracetamol and ibuprofen formulated as tablets were used in patients from seven years old and 82% of evaluations were collected in patients 12 years of age and older. Those medicines were mainly self-administered by the patients. Although used in patients from one year of age, the solution for injection of novaminsulfon was similarly most used in older children (67% ≥ 12 years). Healthcare professionals were in charge of the administration of this medicine. Oppositely, the two medicines formulated as syrup were mainly used in younger children unable to swallow tablets and who needed smaller amounts of active ingredient (64% < 6 years). Furthermore, suppository was used in children up to five years old but mainly in infants and toddlers (97% ≤ 2 years). The last three medicines mainly used in younger children were administered by a caregiver in most cases.

### 3.2. Acceptability

**Figure 1:**
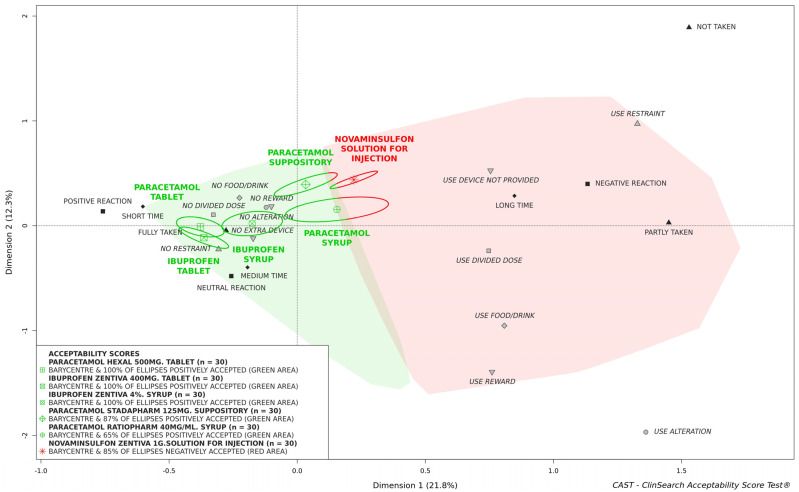
Acceptability scores of the six analgesic/antipyretic medicinal products in pediatrics.

Figure 1 shows the acceptability scores of the six studied medicines, and Table 2 presents the observer-reported outcomes composing these scores.

Both ibuprofen and paracetamol formulated as tablets were similarly well accepted as their barycenters, along with their entire confidence ellipses, were fully located in the green area of the 3D-map. There was no significant difference between both formulations for all the nine observational variables and confidence ellipses overlapped on the 3D-map. The solution for injection of metamizole sodium, similarly mainly used in older patients, was not classified as positively accepted as the barycenter and 85% of confidence ellipses were located in the red area of the map. The difference between acceptability scores of tablets and the solution for injection of metamizole sodium was due to significant differences for four observational variables: the patient’s reaction (χ^2^: *p* < 0.001) was negative for 60% of patients treated with the solution for injection against 5% for those who had taken tablets; the preparation and administration time (χ^2^: *p* < 0.001) which was long for 100% of the evaluations of the solution for injection, while it was short for 68% of the 60 patients who had taken tablet; the use of extra device not provided with the medicine (χ^2^: *p* < 0.001) for administration the solution for injection (e.g., intravenous access, syringes, syringe pump, pipe, needle, minispike); the use of restraint (F: *p* = 0.011) reported only for the solution for injection.

There was a significant difference between the two medicines formulated as syrup: ibuprofen syrup was fully located in the positively accepted profile (barycenter and 100% of confidence ellipses), while 35% of the confidence ellipses surrounding the barycenter of the evaluations of the paracetamol syrup were in the red area. This difference was mainly due to significant differences in terms of the patient’s reaction (χ^2^: *p* = 0.036) and use of an extra administration device (χ^2^: *p* = 0.03). Indeed, the patient’s reaction was negative for 57% of patients who had taken the paracetamol syrup and an extra device was used for 50%, while 27% of the patients had a negative reaction and 20% used an extra device for the ibuprofen formulation. That resulted in a required dose fully taken for 83% of paracetamol syrup evaluations against 93% of evaluations for ibuprofen syrup.

The barycenter of the evaluations of paracetamol suppository was located in the green area, as well as 87% of the confidence ellipses. In this study, this form was mainly used in infants and toddlers (97%). Focusing on the specific subset of patients aged 0 to 2 years, although we can only describe acceptability tendency due to a limited number of evaluations in this subpopulation, suppository appeared to be better accepted than paracetamol syrup, which tended to remain significantly less accepted than ibuprofen syrup (Figure S1). According to Table 2, a high rate of negative reaction (73%) and the use of ointment such as Vaseline—recorded as extra device—(60%) are the main negative components of the acceptability score of paracetamol suppository.

## 4. Discussion

Using the acceptability reference framework, we scored the acceptability of six medicinal products from the drug class of analgesics/antipyretics commonly used at the University Children’s Hospital Düsseldorf. Each score was based on nine observational variables describing the many aspects of acceptability in 30 different individual patients taking the medicine.

In this study, tablets were used in patients from seven years of age and mainly in adolescents aged 12 years and over. Although there are significant differences among children regarding swallowing ability, most of them are able to safely swallow conventional tablet by about six years of age as children at the age of six years have adult-like control during swallowing [26,27]. Swallowing a tablet with water was not considered as using food or drink to help with medication intake. Using the acceptability reference framework, both ibuprofen and paracetamol formulated as tablets were classified as positively accepted. As confidence ellipses overlapped on the 3D-map, there was no statistically significant difference between the acceptability scores of these formulations. Swallowability of tablets might be driven by product’s features such as size, shape, and coating [28]. Although the paracetamol tablet is round (diameter 13 mm, height 5 mm) and the ibuprofen tablet is oblong (length 16 mm, width 8 mm, and height 5.5 mm), both tablets without coating, there was no significant difference in acceptability. Even the individual nine evaluation criteria constituting the reference framework showed no significant difference between paracetamol and ibuprofen tablets. These findings confirmed previous results from a study carried out in Morocco using the CAST methodology, indicating that tablets are accepted in grade-schoolers and adolescents at hospital [18]. Tablets have no need for preparation and a low relative cost.

Such advantages do not apply for the solution for injection, which was also mainly used in grade-schoolers and adolescents in this study. According to the acceptability reference framework, the novaminsulfon 1 g solution for injection was not classified as positively accepted because the barycenter and 85% of confidence ellipses were located in the red area of the map. This was mainly due to negative patient reaction to the placement of the intravenous access as well as preparation and administration process burden. Pain and anxiety are likely to be related to parenteral administration, especially in pediatrics due to a high needle fear prevalence [29]. According to a trained researcher who observed the application of the medicines, the infusion of novalminsulfon itself did not cause any negative reaction led by pain. However, such parenteral formulations allow drug administration for patients who are seriously ill or who have swallowing impairment. Furthermore, this route of administration is suitable for emergency as well as postoperative situations and allows the drug to be absorbed more rapidly and avoid the first-pass metabolism, which might be a clinical requirement.

Oral liquid preparations such as syrup were commonly used in young children unable to swallow conventional SODF such as tablets. Although tablet’s coating provides a physical barrier between the patient’s taste buds and the drugs which are often bitter and aversive to children [6], taste-masking remains challenging for oral liquid preparations and palatability is often an issue. Required volume of medication could be also problematic. Furthermore, while providing a high dose flexibility, there is a need for appropriate measuring and administering device to avoid the risk of incorrect dosing [28]. In this study, ibuprofen and paracetamol syrups were commonly used in children under six years of age. However, there was a significant difference of acceptability between both products: ibuprofen was classified as positively accepted while paracetamol was not. This variation was mainly due to significant difference in term of patient reaction, which is likely to be driven by product taste. Taste of formulations of ibuprofen and paracetamol has been previously studied in pediatrics and both seemed acceptable [30,31,32,33,34,35]. These studies found formulations of paracetamol and ibuprofen to be equally palatable [30,35], or indicated a better palatability of ibuprofen [33,34]. Nevertheless, it is likely that different formulations of both ibuprofen and paracetamol may have different palatability, primarily due to changes in excipients [31,36]. In this study, the better accepted ibuprofen syrup was flavored with strawberry, and the less accepted paracetamol syrup was flavored with orange. Assessing the palatability of analgesic medicines in children, Smith C.J. et al. [33] highlighted that strawberry was mostly reported as the preferred flavor by both genders, while orange was the second worst flavor. A previous study on acceptability of antibiotics in children using the CAST methodology, similarly highlighted that strawberry aroma could be an appropriate option for flavoring oral liquid preparations regardless of patient’s sex [21]. Beyond product characteristics, such as active pharmaceutical ingredient and excipients (e.g., aroma and sweeteners), the device is also likely to impact medicine acceptability in pediatrics [21]. In this study, the better accepted ibuprofen syrup was provided with an oral syringe, while paracetamol syrup was provided with a measuring cup. Previous findings indicated that oral liquids provided with an oral syringe were better accepted than those provided with a measuring spoon [21]. More suitable than measuring spoons or cups for dosing accuracy [37], oral syringes are also more convenient for medicine administration in young children. In the hospital, the bottles of all medications are used for several patients consequently, nurses never used the device provided with the medicine for hygienic reasons. Using an extra device highlights the need to use a device not provided with the medicine due to the lack of device or an unsuitable one. If a device similar to that provided with the medicine was used (e.g., an oral syringe with the ibuprofen syrup), we did not consider this as using an extra device because this was due to hygiene, not due to an inappropriate provided device. In the study, there was a significant difference in term of using an extra device between ibuprofen and paracetamol syrups. Indeed, both syrups were mainly given with a handy oral syringe, rather than a less suitable cup.

In this study, suppositories were also used in infants and toddlers to administer analgesics. Like tablets, suppositories have no need for preparation and a low relative cost. Furthermore, they avoid the first-pass metabolism, do not require taste-masking, and could be administer to children unable to swallow SODF safely [38,39]. However, their acceptability is varying among cultures and according to patient age [26]. In this study, suppositories cannot be classified as positively accepted due to a part of confidence ellipses which failed in the negative area of the reference framework. This was mainly due to a relatively high rate of negative reaction and the need of ointment to ease administration. Improving product design by modifying shape, size, or firmness of suppositories may ease insertion and handling. Nevertheless, it seems to be an alternative to oral liquids with palatability and administration issues, such as the poorly accepted paracetamol syrup, in those very young patients. However, suppositories have varying levels of acceptability around the world depending upon geographic location and culture.

In this study, geographical regions of origin of the child’s parents were recorded. Nevertheless, the influence of cultural background on medicine acceptability cannot be explored conclusively due to a lack of evaluations to get relevant acceptability scores for different geographical regions of origin. Similarly, there was a limited number of evaluations to investigate the influence of age on medicine acceptability. Further evaluations are needed to overcome those limits of the study. Furthermore, comparing acceptability of those medicines in the hospital vs. home setting would be of interest, as previous findings indicated an effect of the context with a greater acceptability at hospital [21].

## 5. Conclusions

These findings highlight acceptability differences among different analgesics/antipyretics in pediatric patients. Differences appear to be driven by dosage forms—e.g., poor acceptability of preparations for injection due to pain in patients as well as due to the preparation and administration process burden; as well as by formulations—e.g., acceptability variations between various syrup formulations. Furthermore, this study highlights the lack of appropriate medicines. These findings improve knowledge on acceptability drivers and might help in formulating and prescribing better medicines for children.

## Figures and Tables

**Table 1:** Characteristics of the 180 patients included in the study, stratified by medicinal product.

Characteristics	Paracetamol 500 mg Tablet (*n* = 30)	Ibuprofen 400 mg Tablet (*n* = 30)	Paracetamol 40 mg/mL Syrup (*n* = 30)	Ibuprofen 4% Syrup (*n* = 30)	Paracetamol 125 mg Suppository (*n* = 30)	Novaminsulfon 1 g Solution for Injection (*n* = 30)
**Sex**						
Female	19 (63) ^a^	15 (50)	11 (37)	13 (43)	12 (40)	11 (37)
Male	11 (37)	15 (50)	19 (63)	17 (57)	18 (60)	19 (63)
**Age group**						
0–2 years	0 (0)	0 (0)	10 (33)	12 (40)	29 (97)	3 (10)
3–5 years	0 (0)	0 (0)	8 (27)	8 (27)	1 (3)	1 (3)
6–11 years	6 (20)	5 (17)	9 (30)	7 (23)	0 (0)	6 (20)
12–17 years	24 (80)	25 (83)	3 (10)	3 (10)	0 (0)	20 (67)
**Parents Geographical regions of origin**						
Western Europe	8 (27)	16 (53)	10 (34)	12 (39)	15 (50)	16 (53)
Central and eastern Europe	5 (17)	3 (10)	7 (24)	5 (17)	3 (10)	5 (17)
Mix including western Europe	6 (20)	2 (7)	3 (10)	5 (17)	5 (17)	1 (3)
Middle east	4 (13)	4 (13)	4 (13)	5 (17)	2 (7)	0 (0)
Northern Africa	4 (13)	0 (0)	1 (3)	1 (3)	3 (10)	4 (13)
Western Europe (no information for other parent)	2 (7)	2 (7)	1 (3)	0 (0)	1 (3)	2 (7)
Other ^b^	1 (3)	3 (10)	4 (13)	2 (7)	1 (3)	2 (7)
**Person in charge of the medicine administration**						
The patient	28 (93)	29 (100)	6 (20)	5 (17)	0 (0)	0 (0)
A caregiver ^c^	0 (0)	0 (0)	16 (53)	15 (50)	22 (73)	0 (0)
A healthcare professional	2 (7)	0 (0)	8 (27)	10 (33)	8 (27)	30 (100)
*Missing data*		1				

^a^*n* (%): number and percentages; ^b^ other: mix without western Europe, sub-Saharan Africa, eastern Asia, central and south America, Indian subcontinent, and central Asia; ^c^ caregiver: family member, other adult helper.

**Table 2:** Observer-reported outcomes collected in this study for the six analgesic/antipyretic medicinal products

Observer Reported Outcomes	Paracetamol 500 mg Tablet (*n* = 30)	Ibuprofen 400 mg Tablet (*n* = 30)	Paracetamol 40 mg/mL Syrup (*n* = 30)	Ibuprofen 4% Syrup (*n* = 30)	Paracetamol 125 mg Suppository (*n* = 30)	Novaminsulfon 1 g Solution for Injection (*n* = 30)
**Result intake**						
Fully taken	29 (97) ^a^	30 (100)	25 (83)	28 (93)	30 (100)	29 (97)
Partly taken	0 (0)	0 (0)	5 (17)	1 (3)	0 (0)	0 (0)
Not taken	1 (3)	0 (0)	0 (0)	1 (3)	0 (0)	1 (3)
**Patient reaction**						
Positive	5 (17)	4 (13)	3 (10)	9 (30)	1 (3)	2 (7)
Neutral	22 (73)	26 (87)	10 (33)	13 (43)	7 (23)	10 (33)
Negative	3 (10)	0 (0)	17 (57)	8 (27)	22 (73)	18 (60)
**Preparation and administration time**						
Short	22 (73)	19 (63)	6 (20)	8 (27)	18 (60)	0 (0)
Medium	6 (20)	10 (33)	15 (50)	18 (60)	8 (27)	0 (0)
Long	2 (7)	1 (3)	9 (30)	4 (13)	4 (13)	30 (100)
**Divided dose**						
No divided dose	26 (87)	25 (83)	25 (83)	26 (87)	29 (97)	30 (100)
Use divided dose	4 (13)	5 (17)	5 (17)	4 (13)	1 (3)	0 (0)
**Food/drink**						
No food/drink	29 (97)	30 (100)	21 (70)	22 (73)	30 (100)	30 (100)
Use food/drink	1 (3)	0 (0)	9 (30)	8 (27)	0 (0)	0 (0)
**Alteration**						
No alteration	28 (93)	25 (83)	30 (100)	30 (100)	30 (100)	30 (100)
Use alteration	2 (7)	5 (17)	0 (0)	0 (0)	0 (0)	0 (0)
**Extra device** ^b^						
No extra device	30 (100)	27 (90)	15 (50)	24 (80)	12 (40)	0 (0)
Use extra device	0 (0)	3 (10)	15 (50)	6 (20)	18 (60)	30 (100)
**Reward**						
No reward	30 (100)	30 (100)	29 (97)	30 (100)	30 (100)	30 (100)
Use reward	0 (0)	0 (0)	1 (3)	0 (0)	0 (0)	0 (0)
**Restraint**						
No restraint	30 (100)	30 (100)	21 (70)	27 (90)	18 (60)	26 (87)
Use restraint	0 (0)	0 (0)	9 (30)	3 (10)	12 (40)	4 (13)

^a^*n* (%): number and percentages; ^b^ device not provided.

## Data Availability

Data underlying the study cannot be made publicly available due to legal and ethical considerations. European Union (GDPR) and French (Law no. 78–17 of 6 January 1978) laws restrict the public sharing of personally identifiable data. Requests for data will be processed according to the French MR-003 Code of conduct by the data controller, ClinSearch, which allows for the use of data for the purpose of reproducing study results. Requests to access the data for this purpose may be sent to the data protection officer of ClinSearch: dataprivacy@clinsearch.net and researchers outside the European Union will need to sign a transfer agreement.

## Supplementary Materials

The following are available online at www.mdpi.com/article/10.3390/pharmaceutics14020337/s1, Figure S1: Acceptability tendency for three analgesic/antipyretic medicinal products in children aged 0 to 2 years.
