# Supplementary material for: Dosage Forms Suitability in Pediatrics: Acceptability of Analgesics and Antipyretics in a German Hospital
Source: Pharmaceutics. 2022 Jan 31;14(2):337. doi: 10.3390/pharmaceutics14020337 (PMC8879646; doi:10.3390/pharmaceutics14020337)
Supplement: Supplementary file 1 [file pharmaceutics-14-00337-s001.zip › pharmaceutics-1554967-supplementary.pdf]

pharmaceutics

Supplementary Materials: Dosage Forms Suitability in Pediatrics: Acceptability of Analgesics and Antipyretics in a German Hospital

Viviane Klingmann, Thibault Vallet, Juliane Münch, Robin Stegemann, Lena Wolters, Hans-Martin Bosse, and Fabrice Ruiz

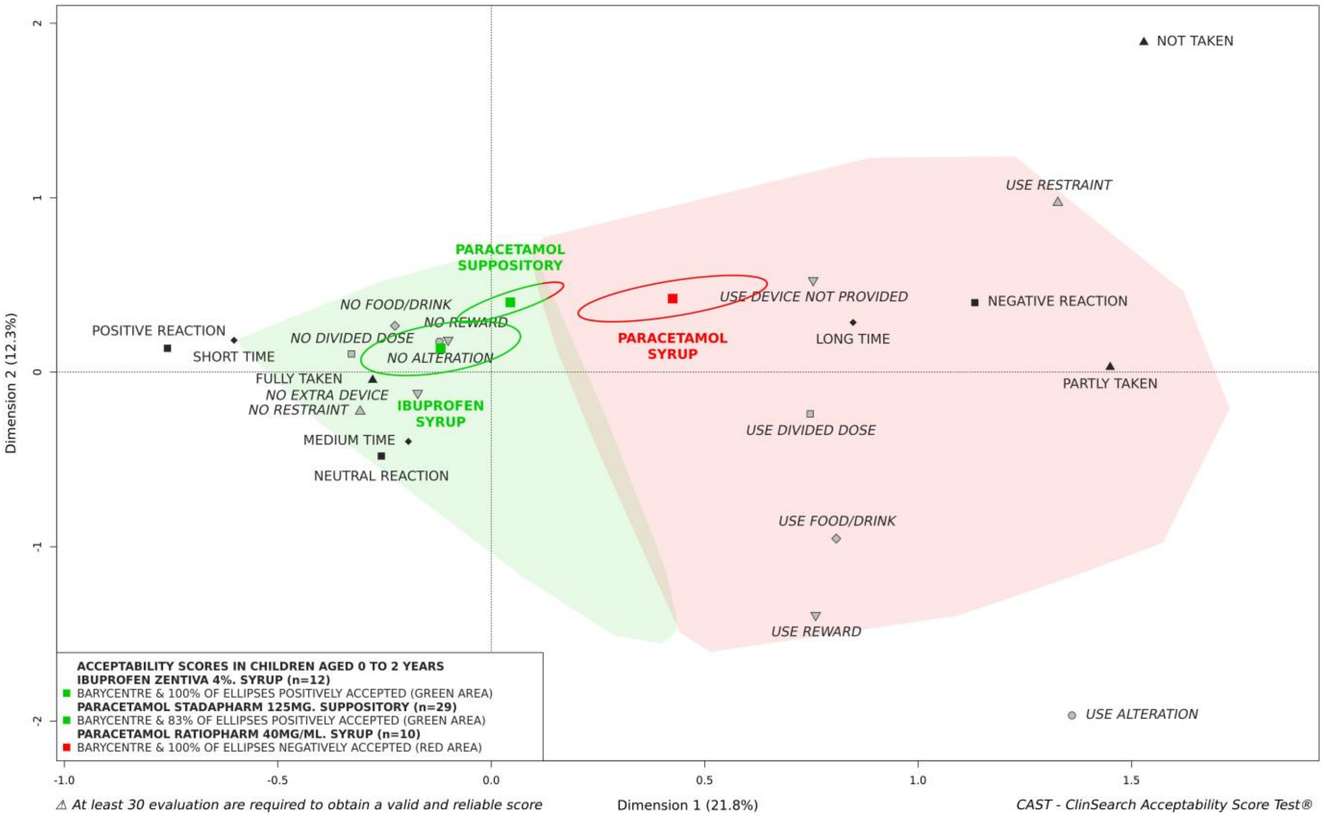

Figure S1. Acceptability tendency for three analgesic/antipyretic medicinal products in children aged 0 to 2 years.
